# Joint micrograph denoising and protein localization in cryo-electron microscopy

**DOI:** 10.1017/S2633903X24000035

**Published:** 2024-03-06

**Authors:** Qinwen Huang, Ye Zhou, Hsuan-Fu Liu, Alberto Bartesaghi

**Affiliations:** 1 Department of Computer Science, Duke University, Durham 27708, NC, USA; 2 Department of Biochemistry, Duke University School of Medicine, Durham 27705, NC, USA; 3 Department of Electrical and Computer Engineering, Duke University, Durham 27708, NC, USA

**Keywords:** cryo-electron microscopy, multitask learning, object detection, semi-supervised learning, single-particle analysis

## Abstract

Cryo-electron microscopy (cryo-EM) is an imaging technique that allows the visualization of proteins and macromolecular complexes at near-atomic resolution. The low electron doses used to prevent radiation damage to the biological samples result in images where the power of noise is 100 times stronger than that of the signal. Accurate identification of proteins from these low signal-to-noise ratio (SNR) images is a critical task, as the detected positions serve as inputs for the downstream 3D structure determination process. Current methods either fail to identify all true positives or result in many false positives, especially when analyzing images from smaller-sized proteins that exhibit extremely low contrast, or require manual labeling that can take days to complete. Acknowledging the fact that accurate protein identification is dependent upon the visual interpretability of micrographs, we propose a framework that can perform denoising and detection in a joint manner and enable particle localization under extremely low SNR conditions using self-supervised denoising and particle identification from sparsely annotated data. We validate our approach on three challenging single-particle cryo-EM datasets and projection images from one cryo-electron tomography dataset with extremely low SNR, showing that it outperforms existing state-of-the-art methods used for cryo-EM image analysis by a significant margin. We also evaluate the performance of our algorithm under decreasing SNR conditions and show that our method is more robust to noise than competing methods.

## Impact Statement

This research paper describes a particle picking and micrograph denoising method for cryo-electron microscopy (cryo-EM) images designed to work under challenging signal-to-noise ratio (SNR) conditions. Based on the idea of multitask learning, we propose to use a deep-learning framework that enables accurate particle localization and micrograph denoising simultaneously. We validate the proposed approach on multiple single-particle cryo-EM datasets and cryo-electron tomography tilted images, showing substantial improvements in SNR and particle picking accuracy. This work is addressed to people working at the interface between biological imaging and computer vision. Advances in denosising and particle identification are important to accelerate and automate the processing of challenging cryo-EM datasets of biomedical significance, such as low-molecular-weight complexes or targets imaged in their native state.

## 1. Introduction

**Figure 1.**
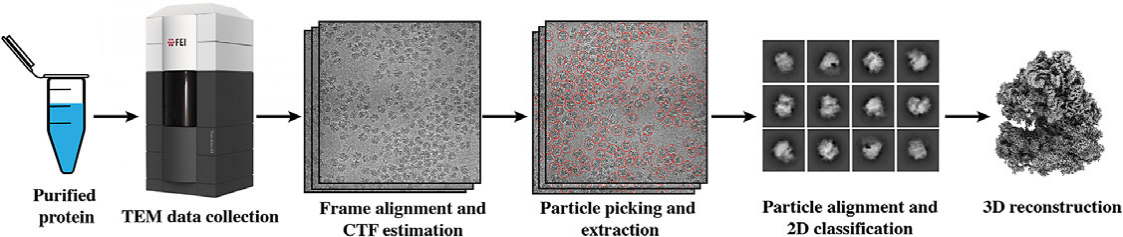
**Single-particle cryo-electron microscopy (cryo-EM) structure determination pipeline**. Proteins are purified, plunge frozen, and subjected to transmission electron microscopy (TEM) imaging. Movie frames of the sample under cryogenic conditions are collected using an electron microscope. Frame alignment and averaging, and contrast transfer function (CTF) estimation are performed as preprocessing steps. For refinement, particles first need to be identified and extracted from micrographs. 2D classification and 3D alignment are performed on extracted particle stacks. With estimated relative orientations, these 2D particles get back-projected into 3D space and a high-resolution reconstruction is obtained.

Cryo-electron microscopy (EM) combined with single-particle analysis (SPA) is a popular technique that enables visualization of proteins and macromolecular complexes at near-atomic resolution.^(^^1^^)^ Data collected using this technique have an extremely low signal-to-noise ratio (SNR), as electron dosage has to be kept low to prevent radiation damage to the biological sample.^(^^2^^)^ Therefore, large amounts of data have to be acquired in order to obtain high-resolution 3D reconstructions. A typical SPA dataset usually contains thousands of micrographs and each micrograph contains a few hundred copies of the protein of interest. To obtain a single high-resolution structure, hundreds of thousands of 2D projections of particles with random orientations need to be detected within micrographs in a process commonly referred to as *particle picking.* Particle projections are then extracted, aligned, and back-projected to obtain a 3D reconstruction (Figure 1). The resolution of the reconstructed 3D structure increases log-linearly with the number of good particles used, making accurate particle picking a critical task for the downstream processing tasks. However, particle detection can be quite challenging due to the low SNR nature of cryo-EM images caused by the limited electron doses used during acquisition to prevent radiation damage. Currently, on datasets of larger-sized proteins with relatively high SNR, particle picking can be performed automatically using either size-based, template matching, or more advanced deep-learning-based algorithms, such as semi-supervised learning-based Topaz^(^^3^^)^ and fully supervised learning-based crYOLO.^(^^4^^)^ On datasets of smaller-sized proteins with extremely low SNR, however, existing automatic picking algorithms are susceptible to under/over picking.^(^^5^^)^ While manual picking can be performed as a last resort, it is laborious and time consuming. As a result, the particle picking of small-sized proteins from images with very low SNR remains a significant bottleneck.

Recent efforts to tackle particle picking under extremely low SNR mainly focus on image quality enhancement through denoising. For example, simple denoising techniques such as low-pass filtering have been widely adopted in the field. Despite its simplicity, frequency cutoff-based approaches have one major drawback: the risk of over-smoothness, which can remove important high-frequency information and make particles indistinguishable from the background. Recent advancements have seen the emergence of neural network-based methodologies that leverage the Noise2Noise (N2N) principle^(^^4^^,^^6^^–^^8^^)^ or noise distribution modeling using generative models on synthetic data.^(^^9^^)^ Methods utilizing synthetic data, however, often yield suboptimal results when applied to real datasets due to their inability to fully capture the true noise distribution. Consequently, N2N-based techniques have gained prevalence since they do not depend on synthetic data. Nonetheless, these methods typically require complex training protocols and the need for paired noisy images, which are usually generated by splitting data movies into even and odd frames (and aggregating them separately), inherently compromising their SNR. This limitation is particularly significant for small-sized proteins where the SNR is already low in the average of all movie frames, and using half the data further diminishes their SNR and consequently the efficacy of N2N denoising. Recent work in natural^(^^10^^)^ and biomedical imaging^(^^11^^)^ has demonstrated that high-quality denoised images can substantially enhance downstream task performance, such as object detection and segmentation. Inspired by these findings, we hypothesize that particle picking, especially under the challenging conditions of low SNR, can be significantly improved if the input micrographs are denoised effectively, ensuring that critical particle features are preserved.

Therefore, to overcome low SNR and expensive data labeling challenges, we propose a framework that enables joint image denoising and particle identification. The information learned from these tasks is complementary and therefore by enabling information sharing, we are able to improve the performance of both tasks. To enable image denoising without ground-truth clean images or paired noisy images, and particle picking with sparsely annotated data, our framework incorporates both self-supervised Bayesian denoising^(^^12^^)^ and positive-unlabeled (PU) learning.^(^^3^^,^^13^^,^^14^^)^ By leveraging the prior distribution of the underlying clean signal, our model learns to denoise without being trained using paired noisy images. The joint learning framework is able to improve the visibility of particles on low SNR micrographs and identify more particles under challenging scenarios while maintaining a low false positive (FP) rate. Training of the framework eliminates the need for splitting movie stacks into two separate sub-stacks for paired denoising training and requires only a few partially labeled micrographs, thus greatly reducing laborious data labeling.

The proposed framework can be applied both to cryo-EM micrographs and cryo-electron tomography (CET) tilt series.^(^^15^^,^^16^^)^ To validate the performance of our approach, we performed experiments on three cryo-EM datasets and one CET dataset. The cryo-EM datasets correspond to one high-molecular-weight target (70S ribosome, 2.5 MDa) and two low-molecular-weight proteins (trehalose-6-phosphate synthase from fungal pathogen *Cryptococcus neoformans* [CnTPS, 280 kDa]) and a sample of aldolase (157 kDa) from EMPIAR-10215.

On the ribosome dataset, we evaluated the performance of our method under different SNR levels. Since micrographs obtained by averaging all movie frames have relatively high SNR, we are able to treat these frame averages as ground truth and use frame averages from subsets of frames to mimic decreasing SNR levels. Using these images, we show that our model is more robust to noise as SNR conditions worsen, with the denoised outputs exhibiting a 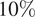 improvement in peak SNR (PSNR) and the detection algorithm producing 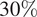 more particles.

For the CnTPS dataset, we labeled a total of 140 particles and were able to produce a set of particles that were missed by other algorithms, which led to a resolution of 3.15 Å after 3D reconstruction.^(^^17^^)^ On EMPIAR-10215, we used a total of 180 labeled particles for training and identified 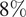 more particles than other approaches, which improved the final 3D resolution from 2.7 to 2.6 Å. We also show that compared to performing denoising and detection sequentially, joint learning is able to achieve better results in both tasks.

On the CET dataset, we show a significant improvement in protein visualization in both raw tilt series and reconstructed tomograms. We validate the detection results by identifying particles from 2D tilt series images, achieving a 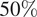 increase in precision and recall scores. For specific tasks that require either denoising or detection alone, the proposed framework also has a mode that can perform these two tasks separately.

To summarize, the main contributions of this paper are as follows:

1. We propose a joint learning framework that allows simultaneous micrograph denoising and particle identification. By leveraging self-supervised Bayesian denoising and PU learning, training of our model does not require ground-truth clean images or paired noisy images, and does not need large amounts of annotated data.
2. Through extensive experimental validation, we show that by performing two tasks simultaneously, we are able to achieve better denoising performance (i.e., increased particle visibility without over-smoothing) and identify particles even under extremely low SNR conditions, thus outperforming existing methods.
3. This method can be applied to single-particle cryo-EM micrographs and CET tilt series for both denoising and detection. Particle coordinates obtained from 2D tilt series can be converted to 3D tomogram coordinates through backprojection and used for sub-tomogram averaging.

## 2. Related work

Recent developments in deep learning have led to breakthrough performance in tasks such as image enhancement, object detection and segmentation. In this section, we introduce relevant recent work, including denoising without clean images, semi-supervised object detection, and multitask learning (MTL).

### 2.1. Denoising without clean images

Unlike denoising algorithms based on supervised learning which are trained using noisy-clean image pairs, blind image denoising is usually achieved by leveraging internal data statistics. Traditional methods based on internal statistics include nonlocal means, which predicts clean pixel values based on similar local neighborhoods,^(^^18^^)^ and block-matching 3D (BM3D), which similarly relies on data repetitiveness.^(^^19^^)^ More recently, denoising methods based on convolutional neural networks have been proposed, including deep image prior which trains a neural network to learn the prior distribution of data from pure noise,^(^^20^^)^ N2N that learns to denoise using pairs of independently corrupted training images that share the same underlying signal,^(^^6^^)^ and Noise2Void, which assumes the independence of noise corruption for each pixel and trains the denoising network only using the single input noisy image by masking the central pixel.^(^^21^^)^ Built on Noise2Void, a more generalized formulation was proposed in Reference (22) and was further improved by incorporating Bayesian statistics in Reference (12). In addition, a number of related image denoising methods based on deep learning have been proposed.^(^^23^^–^^27^^)^ For the case of cryo-EM images, implementations of N2N that also provide generalized pre-trained models for image denoising have been successfully applied.^(^^7^^,^^28^^)^

### 2.2. Semi-supervised object detection and its application in cryo-EM

Built upon supervised object detection frameworks,^(^^29^^–^^32^^)^ semi-supervised methods that incorporate the use of unlabeled data through pseudo-labeling and consistency regularization, along with supervised learning of labeled data, have been proposed.^(^^33^^–^^35^^)^ In Reference (36), data augmentation is applied and the model is trained through maximizing consistency between detection and classification outputs of labeled/unlabeled images and their augmented pairs. Sohn et al.^(^^37^^)^ proposed a semi-supervised framework that use labeled data to first train a teacher model and then update the model by maximizing the consistency between output pseudo-labels of unlabeled data and strongly augmented pairs. To alleviate the problem of confirmation bias and further improve the quality of pseudo annotations, Feng Zhou et al.^(^^38^^)^ proposed an adoption of instant teacher that uses instant pseudo labeling with extended weak-strong data augmentations for teaching during each training iteration, instead of training in a sequential manner. In the cryo-EM field, several deep learning-based object detection methods have been used successfully for particle detection.^(^^3^^,^^4^^,^^8^^,^^39^^,^^40^^)^ crYOLO^(^^4^^)^ is a fully supervised picking method trained with a variety of annotated data built upon the popular YOLO model. Topaz^(^^3^^)^ is a semi-supervised particle picking method based on PU learning that aims to minimize the difference between empirical data distribution and its prior. While generalized pre-trained models are available both in crYOLO and Topaz, these tend to underperform when applied to lower SNR datasets where the data distribution deviates from the distribution present in the training set.

### 2.3. MTL and object detection

While neural networks have shown impressive results for various tasks such as the aforementioned denoising and detection, these tasks are solved in isolation, that is, a different network is trained for each task. Recently, MTL which aims to train a single network to perform different tasks simultaneously has shown promising results.^(^^41^^–^^47^^)^ MTL leverages complementary information shared between related tasks to improve model generalization and the performance of the original task. Associated tasks can also act as regularizers for one another. Earlier applications of MTL in object detection include Mask-RCNN, which extends the Faster R-CNN^(^^48^^)^ by adding a branch for predicting segmentation masks for each object detected in an image. More recently, object detection tasks are often combined with other tasks such as estimating distance between detected objects,^(^^49^^)^ using segmentation masks as attention,^(^^50^^)^ using attention mechanisms to dynamically weigh the contributions of different tasks during training and inference,^(^^51^^)^ and performing denoising and detection through a cascaded network in a supervised manner.^(^^52^^)^ Inspired by these MTL applications, Buchholz et al. combined denoising and supervised segmentation and applied the joint model to fluorescence microscopy images.^(^^53^^)^ Most of these strategies operate on images with a relatively high SNR and require full supervision on at least one task. In earlier versions of our work,^(^^54^^,^^55^^)^ there have also been advances in joint learning techniques applied to lower SNR cryo-EM images that require less supervision. However, these methods have limitations, as they either rely heavily on pixel intensity assumptions or perform poorly when applied to images of crowded environments. Building on the success of these methods, we seek to extend their applicability to lower SNR datasets such as those obtained from SPA and CET modalities of cryo-EM.

## 3. Method

**Figure 2.**
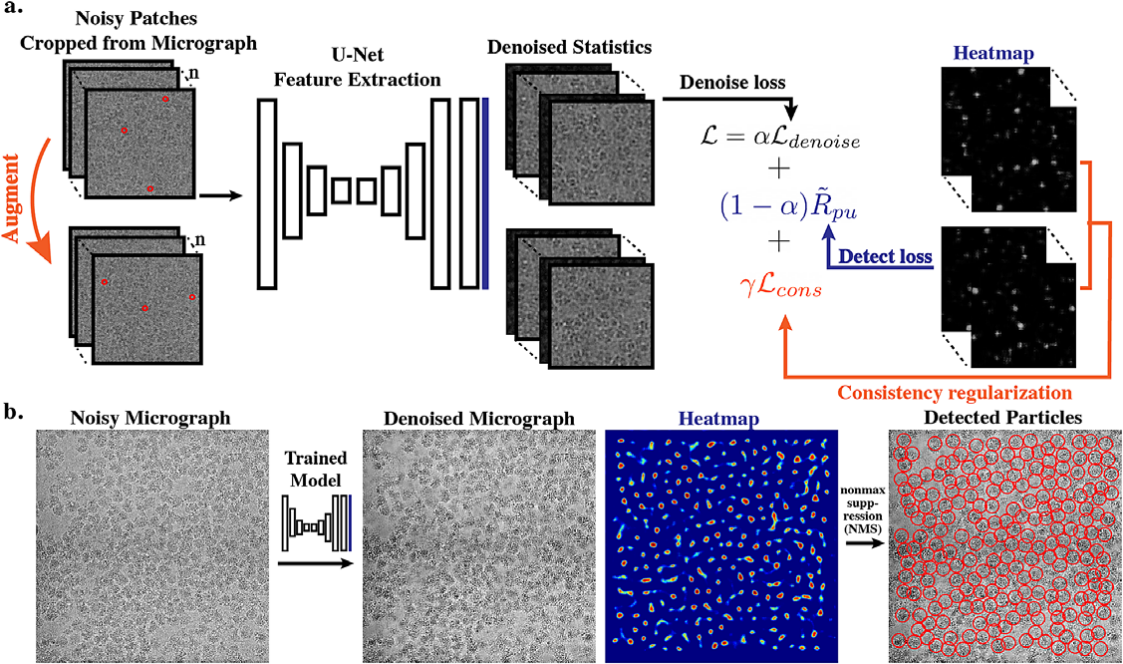
**Main workflow of our proposed framework**. (a) Training of the network utilizes image patches cropped from the partially labeled input micrographs. Image patches and their augmented pairs first go through a feature extraction backbone. Outputs of the backbone go into both the denoising head and the detection head (black). The denoising head outputs the estimated statistics of the underlying clean signal and the detection branch outputs the probability of a pixel being at the center of a particle. Consistency regularization is applied to outputs from the original input and the augmented pairs. (b) For the evaluation workflow, noisy images are fed into the network and clean images and their corresponding detected particles are generated.

As shown in Figure 2, the proposed framework is composed of three main components: (i) a U-Net-based feature extraction backbone; (ii) a denoising head that outputs the clean image and a detection head that outputs the probability of a pixel being at the center of a particle; and (iii) an auxiliary noise estimation network that estimates the probability distribution of the noise. Non-max suppression is applied to the detection head output to get the final particle coordinates. If only denoising or detection is needed, the other head can be easily turned off so that the framework performs only a single task. During the training stage, cropped patches of noisy micrographs are fed into the network. During the inference stage, the network takes in the whole micrograph and outputs a denoised image and a heatmap used for particle localization.

### 3.1. Denoiser training using a single noisy micrograph

The denoiser is trained based upon a blindspot convolutional neural network proposed in Reference (12) that learns the underlying clean signal by maximizing the posterior probability of the observed noisy signal. Denote the underlying clean pixel value as 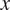 and the observed noisy pixel value as 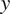 . As pixels are not independent, we assume that 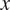 not only depends on 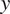 , but also its surrounding context, which we denote as 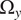 . Specifically, we model the dependence of 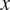 and its surrounding context 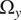 as 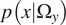 . In a convolutional neural network setting, 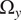 can be viewed as the receptive field surrounding the center pixel 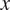 . If we assume noise is independent between pixels and independent of the context, we can model the noise distribution 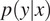 explicitly. With this, we can connect the observed noisy data to the unobserved clean signal using the noise model 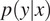 and the signal model 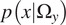 through marginalization:(1)

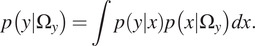

Specifically, we assume the noise model 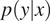 to be Gaussian or Poisson (as commonly assumed in cryo-EM applications). We model the prior 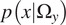 as Gaussian 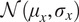 as well, so that the marginal likelihood 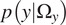 can be computed in closed form. Note that since all micrographs are grayscale images, the prior is a univariate Gaussian. Finally, we are able to infer 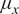 and 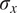 by maximizing the data likelihood under Equation 1. Once we approximate the prior statistics, the final denoised image incorporates the observed noisy signal information through Bayesian reasoning. The posterior 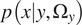 is proportional to the product of the noise model 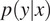 and the prior 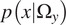 :(2)

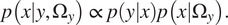

With this, the denoised image can be obtained by calculating the maximum a posteriori estimate.

As mentioned above, our proposed model supports both Gaussian noise and Poisson noise assumptions. Next, we discuss how our model adapts to these noise models.

#### 3.1.1. Gaussian noise model

Since we model the signal corruption as zero-mean Gaussian noise,^(^^56^^)^ the noise model 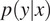 can be expressed as a normal distribution 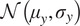 where:(3)

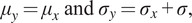

with 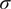 being the standard deviation of the noise which is learned through an auxiliary noise estimator. For simplicity, we decided to use the same backbone architecture (UNet) for this purpose. However, a simpler architecture with fewer parameters such as a multilayer feed-forward convolutional neural network can also be used. Next, we minimize the negative log-likelihood of the observed training data:(4)

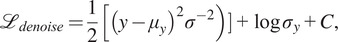

where 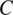 is a constant that can be ignored. Once the prior statistics are learned, we obtain the denoised image using the posterior mean:(5)

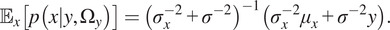



#### 3.1.2. Poisson noise model

While in most cryo-EM applications, the noise is assumed to be Gaussian, a more accurate assumption is to use a Poisson distribution parameterized by the electron count 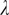 . Therefore, we model the noise distribution as 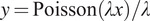 and approximate Poisson noise as signal-dependent Gaussian noise.^(^^57^^)^ In this case, the resulting noise standard deviation is 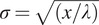 and the noise model becomes:(6)

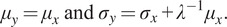

We substituted the above model into the loss function in Equation 4 and the posterior mean in Equation 5, where 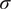 becomes 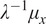 and 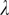 is learned using the auxiliary noise estimator.

This approach allows us to train the network without the need of paired noisy images, which reduces the time needed to divide the aligned cryo-EM movie stacks into two parts. It also reduces potential cumulative errors originating from frame alignment as one of the principles of N2N is that the underlying clean signal has to be the same for both noisy inputs. This condition is hard to satisfy in practice due to the low SNR of cryo-EM frames, which results in inaccurate image alignments.

### 3.2. Detector training using a partially annotated micrograph

The particle detector produces a center point heatmap 
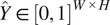
 , where 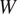 and 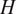 are the width and height for the input micrograph. We extend the method used by Zhou et al.^(^^58^^)^ to generate the ground-truth heatmap 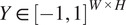 using the partially annotated micrograph. For each annotated center coordinate position 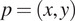 , we apply a Gaussian kernel 
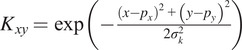
 , where 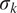 is determined by the particle size based on Reference (59). The remaining unlabeled coordinates on 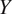 have a value of 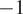 . With such formulation, the detector learns to classify each pixel 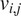 at position 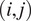 to the corresponding 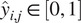 . Denote 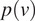 as the underlying data distribution from which 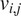 is sampled from. Denote 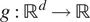 as an arbitrary classifier that can be parameterized by a neural network, and 
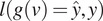
 as the loss incurred when the model outputs 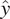 when the ground truth is 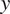 . If all the pixels are labeled, this is essentially a binary classification problem that can be optimized using a standard positive–negative learning approach with the following risk minimization function:(7)

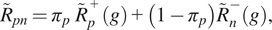

where 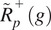 is 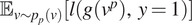 and can be estimated as 
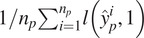
 , and 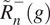 is 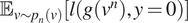 which can be estimated as 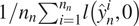 , with 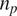 and 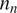 being the number of positive and negative pixels, respectively. In the partially annotated case, only a few positive pixels are labeled (the remainder of the data is unlabeled), which allows us to reformulate the problem into the PU setting. The positive labeled pixels are sampled from 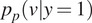 and the remaining unlabeled pixels are sampled from 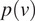 . The classifier 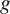 should learn to classify labeled positives, and the empirical distribution of output probabilities of unlabeled pixels should follow the prior distribution 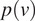 , that is, 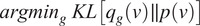 . We can therefore rewrite the risk minimization expression in the PU setting as follows:(8)

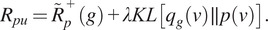

As suggested in Reference (3), we model 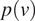 as a binomial distribution parameterized by 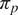 , which represents the probability of observing a particle. For a mini-batch that contains K samples (pixels):(9)

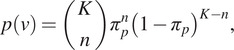

where 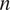 is the number of positive samples. The empirical distribution of the number of positives in the unlabeled samples from the mini-batch obtained from the classifier 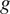 is:(10)

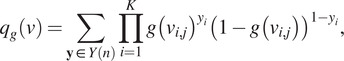

with 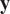 being the indicator vector with 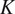 elements, where 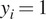 for positive data and 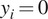 for negatives, and 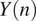 denotes the collection of all such vectors with 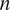 positive data points in total. By substituting 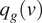 and 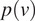 using Equations 9 and 10, we can therefore rewrite Equation 8 as follows:(11)

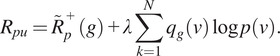

In practice, since the ground-truth heatmap is splatted with Gaussian kernels, the labels are not strictly binary. Therefore, we adopt a pixel-wise logistic regression with focal loss for 
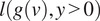
 . Specifically, we have:(12)

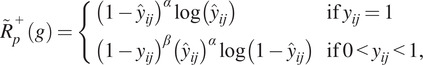

where 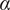 and 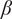 are the focal loss parameters. We use 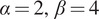 in our experiments.

### 3.3. Overall training objective

In addition to self-supervised denoising and pixel-level PU learning, we imposed consistency regularization to further improve detection performance. For each noisy input 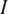 , we randomly apply horizontal and vertical flipping, and 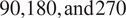 degree rotations to generate augmented pairs 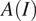 , where 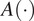 denotes the applied transformation. The augmented images are fed into the same network and the network outputs the denoised statistics and detection heatmap. We assume equivariance in the heatmap, that is, 
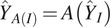
 . Therefore, to impose consistency regularization, we define the consistency loss as follows:(13)

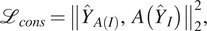

where 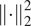 denotes the L2 norm error. The final training objective for the joint framework is thus:(14)

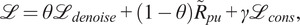

where 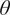 is the weight for each task and 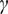 is the weight used for consistency regularization.

### 3.4. Implementation details

#### 3.4.1. Model architecture

We adopt a U-Net-based architecture for the feature extraction backbone that uses a shifted convolutional layer, as proposed in Reference (12) (instead of a normal convolutional layer). The shifted convolutional layer creates a restrictive receptive field that does not include the center pixel. The model contains three encoding layers (downsampling) and three decoding layers (upsampling) with skip connections. For smaller-sized proteins, it is beneficial to use a shallow architecture so that finer details can be learned. The noise estimation network is implemented using the same U-Net architecture, but using standard convolutional layers.

#### 3.4.2. Training of the network

During training, patches of size 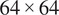 that contain particles are used as input to the network. Random horizontal and vertical flipping are applied as data augmentation. We use a batch size of 16. In order to prevent training instability and over-fitting, we adopted two strategies: (i) learning rate warm up and (ii) early stopping. We start learning with a zero learning rate and gradually increase the learning rate to 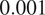 for the first 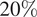 of training iterations. After reaching the initial rate, we adopted cosine decay scheduler that progressively lowers the rate down until the end of training. Early stopping is used when there is less than 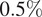 of change in the overall loss. We experimentally found out that 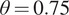 , 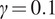 , and 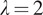 work well and we adopt the ADAM optimizer. Training with 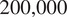 iterations takes less than an hour, and usually the model converges within 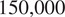 iterations. The model is trained on a single NVIDIA Tesla V100 GPU with 32GB of RAM.

#### 3.4.3. Inference of the network

Given a trained joint model, we perform image denoising and particle detection on full-size micrographs. Given the trained model, inference takes less than a second on each micrograph. To obtain the final particle coordinates, non-maximum suppression is applied. The radius of non-maximum suppression is determined based on the half major-axis length of the particles.

## 4. Results

### 4.1. Description of datasets

We evaluate our methods on one cryo-EM dataset of K63 ribosomes (high-molecular weight), two challenging low-molecular-weight cryo-EM datasets: trehalose-6-phosphate synthase from fungal pathogen *C. neoformans* (CnTPS) and aldolase from EMPIAR-10215, and one CET dataset of 70S ribosomes from EMPIAR-10304.

#### 4.1.1. K63 ribosomes

This dataset contains 1,000 movies with defocus values ranging from 0.8 to 3.0 μm. The pixel size is 1.08 Å. Each movie contains 60 frames. Full frame averages have relatively high SNR as ribosomes have high molecular weight. In order to simulate increasingly lower SNR conditions, such as those observed for lower molecular weight proteins and lower defocus datasets, we use partial averages calculated from 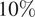 , 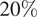 , and 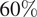 of the total number of frames (6, 12, and 36 frames, respectively). We treat full frame averages as “pseudo clean images” for the denoising task and particles identified using these averages as pseudo ground truth. Movies are motion corrected using MotionCor2.^(^^61^^)^ We used 16 micrographs for training and the remaining ones for testing. Each micrograph is labeled with 20–40 particles. The total training set is composed of 500 particles, which accounts to around 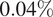 of the total number of particles in the dataset. Micrographs are downsampled by a factor of 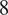 from 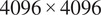 to 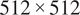 and no exposure weighting was used.

#### 4.1.2. Trehalose-6-phosphate synthase from fungal pathogen C. neoformans (CnTPS)

This dataset contains 3,177 movies with defocus values ranging from 0.5 to 2.5 μm, each of size 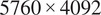 pixels. Movies were collected at a nominal magnification of 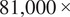 using a pixel size of 1.08 Å. Each movie contains 60 frames. As the dataset is of low-molecular weight and already has low SNR, we used the full frame averages in this case. We followed the same motion correction and downsampling procedure (4× binning) as in the K63 ribosome dataset. The entire training set is composed of 140 labeled particles selected from 2 micrographs.

#### 4.1.3. Rabbit muscle aldolase (EMPIAR-10215)

This dataset of rabbit muscle aldolase contains 1,052 movies with defocus values ranging from 1 to 2 μm, each of size 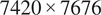 pixels. Movies were collected in super-resolution mode using a pixel size of 0.416 Å. Similar to CnTPS, we used full frame averages for both denoising and detection tasks. We followed the same motion correction and downsampling procedure (4× binning). The training set was composed of 180 labeled particles selected from 2 micrographs.

#### 4.1.4. 70S ribosomes (EMPIAR-10304)

This CET dataset consists of 12 tilt series from a sample of purified 70S ribosomes. Each tilt series is composed of 41 projection images ranging from −60° to +60°. A single tilt image has a size of 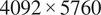 pixels, with a pixel size of 2.1 Å. We downsampled all tilt images by a factor of 8 and performed both tasks on the binned images. The training set for detection is composed of 211 particles selected from 3 images at zero degree tilt.

### 4.2. Joint training improves micrograph SNR and particle visibility

First, we assessed the ability of joint training to denoise images from all datasets and compare our method against BM3D and Topaz denoise. For Topaz denoise (both for 2D micrographs and 3D tomograms), in order to achieve optimal performance, we followed the guidelines in the tutorial (https://github.com/tbepler/topaz) and trained dataset-specific models for each dataset. As model training requires pairs of noisy images, we used the aligned stacks from MotionCor2^(^^60^^)^ and divided the stack into even and odd stacks based on the frame number. The frame averages are obtained from each stack and then fed into the network for training. We used the same training sets for both denoising and detection. For the K63 ribosome dataset, we treat the full dose frame averages as ground-truth clean images and use PSNR values to evaluate denoising performance. As full dosage micrographs can still be noisy, we apply a low-pass filter to remove potential high-frequency noise. As shown in Table 1, the left half shows the mean and standard deviation of PSNR values computed against full dosage frame averages and the right half shows values calculated against low-pass filtered images. Since low-pass filtering can remove high-frequency noise as well as information, we expect the actual PSNR value to lie in between the two reported values. As SNR decreases, our joint learning model is able to significantly outperform other methods. Additionally, joint learning with consistency regularization performs better than learning without consistency across all SNR levels. Overall, neural network-based methods perform better than the traditional denoising method BM3D, especially at lower SNR levels. In particular, we can confirm that at the lowest SNR, our denoising method based on Reference (12) performs much better than Topaz denoise. It is worth pointing out that inputs to Topaz denoise have even lower SNR because training of the network requires splitting the noisy images into odd and even subsets of frame averages.

**Table 1.** Denoising performance (PSNR) for averages obtained from increasing frame fractions compared to the full exposure (with and without application of low-pass filtering). Highest PSNR values for each column are indicated in boldface.

Compared against	Full dose image			Low-pass filtered		
Method	60% dose	20% dose	10% dose	60% dose	20% dose	10% dose
BM3D	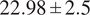	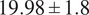	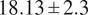	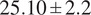	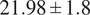	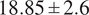
Topaz denoise	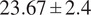	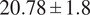	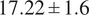	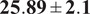	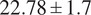	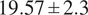
Topaz denoise (pre-trained)	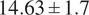	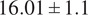	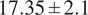	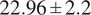	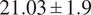	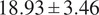
Ours denoising only	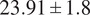	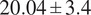	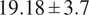	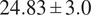	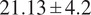	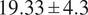
Joint learning without consistency	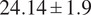	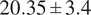	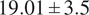	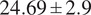	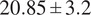	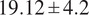
Joint learning with consistency	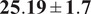	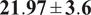	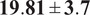	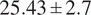	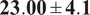	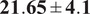

Our results also confirm that the performance of the N2N-based methods is limited by the input noise levels. When input images have extremely low SNR, the network is not able to fully distinguish real signal from noise, thus decreasing the probability of learning the correct noise distribution from pairs of noise-corrupted images. At lower SNR, our joint training method with consistency regularization also performs better than the pure denoising method, further demonstrating that the information obtained from the detection task can be used to improve the denoising task. It should be noted that pure denoising has better performance than joint learning without consistency. This shows the importance of consistency regularization: information shared between multiple tasks is beneficial if properly regularized. Otherwise, naive information sharing can affect the performance negatively. In addition, we observed that using smaller batch sizes can sometimes benefit the denoising task, whereas bigger sizes can improve the performance of the detection task but worsen the denoising performance. With consistency regularization, the influence of the batch size on both tasks is mitigated.

At higher SNR, our joint learning method represents less of an advantage and only performs slightly better than or similar to other methods. This shows that the benefits of the joint learning approach are most noticeable at lower SNRs where the information from the different tasks is successfully aggregated. Conversely, when a sufficient amount of information is present, complementary information from additional tasks is less useful. In addition, since our method incorporates information from the noisy input during the inference process, compared to both low-pass filtering and Topaz denoising, our approach is able to avoid over-smoothness while preserving more structural information.

**Figure 3.**
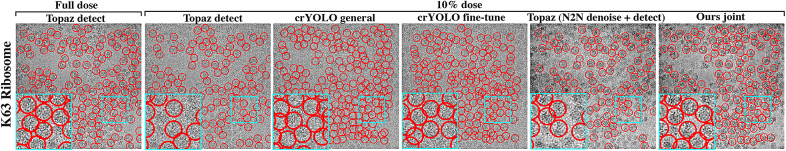
**Qualitative evaluation using a K63 ribosome dataset.** We show one full dose micrograph with particles identified using Topaz, and particle picking results when using micrographs obtained from 10% of the dose using: Topaz, generalized crYOLO model with built-in low-pass filtering, fine-tuned crYOLO model with built-in low-pass filtering, Topaz denoise followed by Topaz picking, and our proposed joint framework. Subregions from the micrographs are highlighted in cyan and particles are highlighted in red. Our method achieves better particle visibility after denoising and identifies more particles and less false positives despite the challenging SNR conditions.

**Figure 4.**
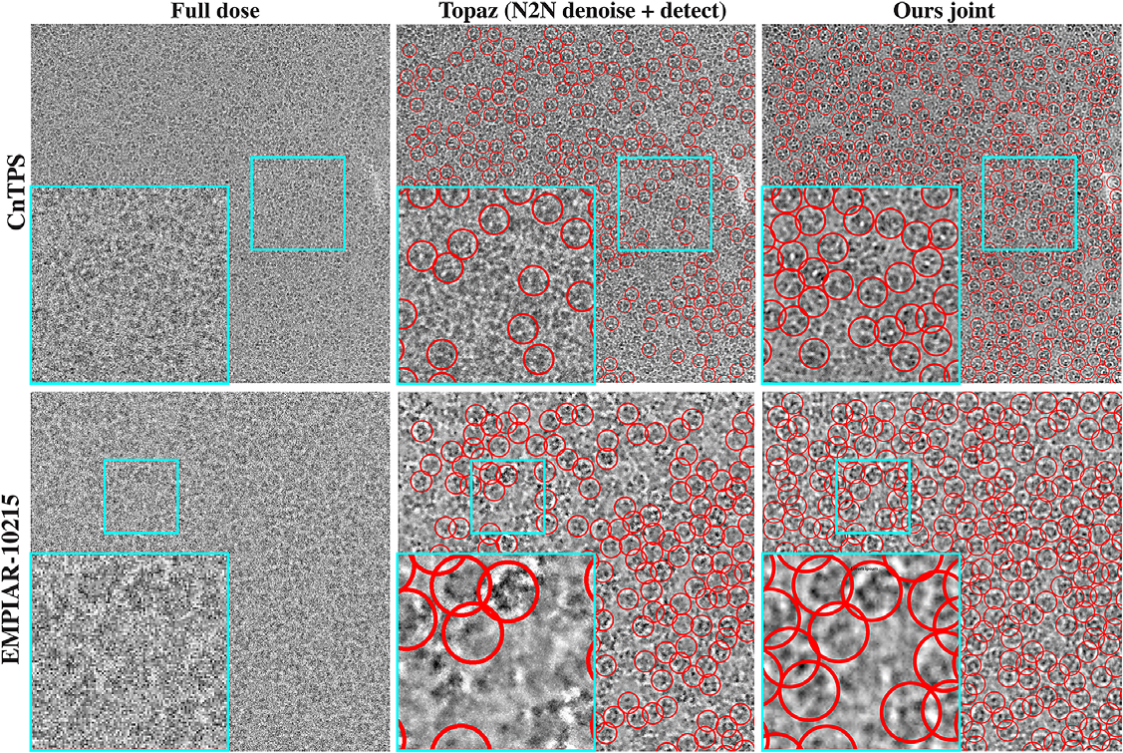
**Qualitative evaluation on CnTPS and EMPIAR-10215 datasets.** Since micrographs have lower SNR in this case, we only show the full dose micrograph, picking results using Topaz denoise followed by Topaz picking, and our joint framework. Subregions from the micrographs are highlighted in cyan and particles are highlighted in red. Similar to the K63 ribosome dataset, our method is able to improve particle visibility which in turn improves particle identification.

For the remaining datasets, as clean images do not exist and the main purpose of image denoising is to facilitate downstream particle identification process by enhancing contrast between background and particle, instead of calculating quantitative measurements such as PSNR, we provide qualitative evaluations, that is, whether particles become more visible after denoising. As shown in Figures 3 and 4, for cryo-EM single-particle micrographs from K63, CnTPS and EMPIAR-10215, our proposed solution is able to better preserve high-frequency particle information without over-smoothing.

**Figure 5.**
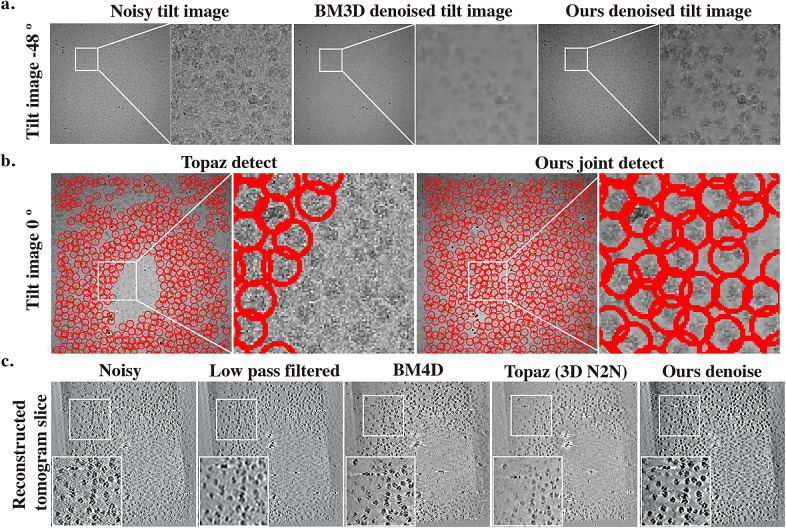
**Qualitative evaluation of denoising and detection on cryo-electron tomography (CET) dataset (EMPIAR-10304)**. (a) Images from the original noisy tilt series and the denoised image using block-matching 3D (BM3D) and our method. Compared to BM3D, our method better preserves features while smoothing the background. (b) With better feature preservation, our method is able to identify more particles on tilted images, compared to directly picking on the noisy raw tilted image. (c) Slices from the raw tomogram, low-pass filtered, BM4D filtered, Topaz denoised using the pre-trained model and ours denoised. Low-pass filtering, BM4D, and Topaz are applied directly on the 3D tomogram, while our 3D tomogram is reconstructed using denoised 2D tilt series shown in a. Similarly, low-pass filtering, BM4D, and Topaz denoising show strong smoothing effects, while ours is able to achieve a better balance between background smoothing and feature preservation.

For the CET dataset (EMPIAR-10304), we provide qualitative evaluations for both the denoised tilt series and 3D tomograms reconstructed using denoised tilt series (Figure 5). After obtaining affine transforms from tilt series alignment using fiducial-based alignment in IMOD,^(^^61^^)^ 3D tomogram reconstruction is performed using IMOD’s backprojection algorithm. The model is able to smooth the background noise while preserving the structural features in all cases. Figure 4 shows that for low-molecular weight-particles that are barely visible on raw micrographs, after denoising, the particles become more visible. Similarly, for CET datasets, there is substantial contrast improvement for tomograms reconstructed using denoised tilt series, where reconstructions show a smooth background.

### 4.3. Joint training improves particle identification in challenging datasets

Next, we assess the ability of the proposed joint framework to detect particles under low SNR conditions and in densely populated environments. In addition, we compare the particles picked using our method with Topaz and crYOLO (K63 ribosome and EMPIAR-10304 datasets only). Since both our proposed framework and Topaz require a few annotated positive particles for training, we used the same training set in all the experiments except for the CnTPS dataset. In this case, we have to use more labeled particles (280 particles) to train Topaz, because the model trained using the original labels was unable to produce a sufficient amount of particles.

For Topaz, instead of fine-tuning the generalized model using new annotated data from all datasets, we trained data-specific models from scratch and adjusted the hyperparameters based on the instructions provided in Reference (3). It is worth noting that because our datasets are very densely populated with irregular-shaped particles, we encountered difficulties with convergence during training when we attempted to fine-tune the pre-trained model.

For crYOLO, we evaluated picking performance using the following: (i) the pre-trained generalized model (https://cryolo.readthedocs.io/en/stable/tutorials/) with crYOLO’s built-in low-pass filtering (crYOLO column in Table 2), (ii) a fine-tuned model trained using our annotations with crYOLO’s built-in low-pass filtering (*crYOLO Fine-Tune* column in Table 2), and (iii) a fine-tuned model using our denoised micrographs as input (*crYOLO with our denoise* column in Table 2). As crYOLO is a fully supervised algorithm and training data require specifying a bounding box size in addition to particle locations, we manually annotated different sets of micrographs and particles. Specifically, for K63 ribosomes, we manually annotated three micrographs, which included 500 particles in total. The newly annotated data were used as training data for model refinement.

**Table 2.** Detection performance on K63 ribosome and EMPIAR-10304 datasets. Highest values for each column are indicated in boldface.

	K63 ribosome			EMPIAR-10304		
	Precision	Recall	AUPRC	Precision	Recall	AUPRC
Topaz detect only	0.867	0.23	0.55	0.48	0.67	0.54
Topaz denoise+detect	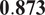	0.27	0.58	N/A	N/A	N/A
crYOLO general model	0.305	0.31	0.28	0.259	0.146	0.12
crYOLO fine-tuned	0.573	0.4	0.45	0.341	0.221	0.21
Ours sequential	0.823	0.41	0.59	0.605	0.443	0.57
Ours detect only	0.814	0.33	0.57	0.584	0.44	0.561
Ours joint	0.835	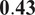	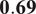	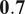	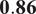	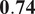

We evaluated detection performance at both 2D and 3D levels. For K63 ribosomes and EMPIAR-10304 that have ground-truth annotations, 2D results are evaluated using precision, recall, and precision–recall area under curve, as shown in Table 2. For the K63 ribosome dataset, we compared the particles identified using 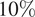 of frames against ground-truth particles identified using full dose frame averages in terms of precision and recall scores and the total number of good particles after 2D classification. For EMPIAR-10304, scores are obtained by comparing against manual labels. We treat particles picked using the full dose as ground truth. True positives (TP) correspond to particles that are detected on both full dose and partial dose micrographs. FPs correspond to particles that were identified on partial dose but not on full dose micrographs. False negatives (FNs) are particles located on full dose but not on the averages obtained from the partial dose micrographs. To account for small variations in the detected particle centers, instead of looking at a single pixel, we also look at pixels around the center pixel within a certain radius. For example, suppose a particle is detected on a full dose micrograph; if the particle is also detected on the partial dose image within a radius of 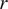 , then the two particles are considered as a true positive match. We use a radius of 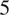 pixels. This way, precision and recall are calculated as follows:(15)

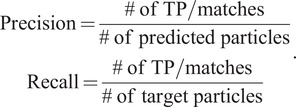

For CnTPS and EMPIAR-10215, as ground-truth labels for the whole dataset do not exist, in 2D, the performance is evaluated only in terms of the total number of good particles after 2D classification and the percentage of good particles. In 3D, the performance is evaluated using the final resolution of the 3D reconstruction. Final resolution is determined using the Fourier shell correlation (FSC), which measures the normalized cross-correlation coefficient between two independently determined reconstructions, each based on half of the particles in the dataset:(16)

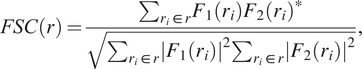

where 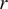 represents a frequency shell in Fourier space, and 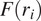 is the Fourier component at the 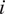 th shell. All processing steps (2D classification, 3D reconstruction, and map sharpening) were performed using the cryoSPARC software.^(^^17^^)^

#### 4.3.1. K63 ribosome

Similar to the denoising task, we found that as the SNR increases, all methods tend to produce similar results both in terms of precision–recall values and total number of clean particles picked. At the lowest SNR ( 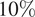 frame averages), however, our joint learning method with consistency regularization is able to perform much better than others. At a cutoff probability threshold of 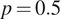 (if the probability of being a particle is greater than 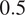 , we treat is as a particle), our method is able to obtain precision, recall, and number of good particles of 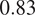 , 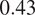 , and 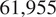 , respectively, compared to 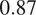 , 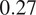 , and 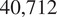 from Topaz, and 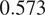 , 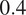 , and 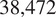 from crYOLO’s fine-tuned model. crYOLO tends to identify more FPs compared to Topaz and our algorithm. Both crYOLO’s generalized model with low-pass filtering and crYOLO’s fine-tuned model using our denoised micrographs were not able to perform well. As both denoised micrographs and 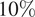 frame averages are very different from micrographs used for training crYOLO’s generalized model, the pre-trained model is not able to generalize well to unseen data from a different data distribution.

#### 4.3.2. EMPIAR-10215

After 2D classification and filtering, 529,212 clean particles from an initial set of 797,229 particles picked by Topaz were selected. For our approach, 574,073 good particles were identified from an initial set of 851,882 positions. Similar to CnTPS, clean particles obtained from 2D classification were used for further processing with fixed seeding. Ab initio reconstruction was performed in cryoSPARC followed by eight iterations of nonuniform refinement using D2 symmetry. Particles selected using our method resulted in a reconstruction at 2.6 Å resolution (using the 0.143-cutoff FSC criteria), compared to 2.7 Å obtained using particles picked by Topaz.

#### 4.3.3. CnTPS

**Figure 6.**
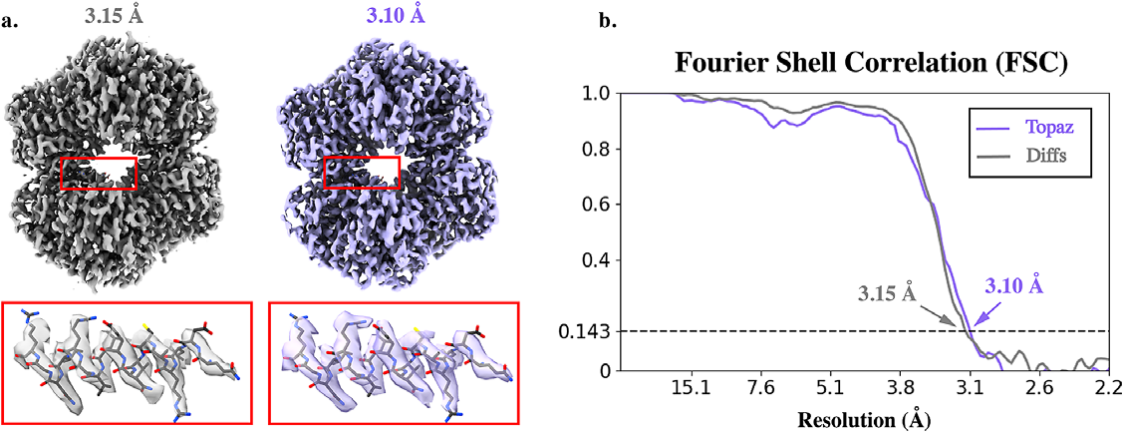
**Comparison of 3D reconstructions of CnTPS obtained using particles produced by Topaz and our method**. (a) Overall reconstructions and secondary structure features corresponding to the areas highlighted in red is shown with atomic model in wire-frame representation fit into the density. Map produced with particles picked by our approach and missed by Topaz (gray) and map produced using particles picked by Topaz. (b) Corresponding Fourier shell correlation (FSC) curves obtained from particles picked by our approach with Topaz particles removed (gray, Diffs) and using particles picked by Topaz (purple), showing that particles picked by our algorithm (and missed by Topaz) still produce a high-resolution reconstruction.

After 2D classification and filtering, 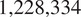 clean particles were left from an initial set of 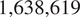 positions picked by Topaz. Our approach resulted in 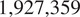 good particles from a total of 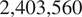 starting positions. As shown in Figure 6, despite using fewer particles for training, our method is able to identify more good particles while keeping a low FP rate (ours 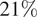 vs. Topaz 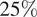 ). We then compared the quality of each particle set through 3D reconstruction. For each set of particles, clean particles obtained from 2D classification were used for further processing using fixed seeding. We confirmed that the 2D class averages showed the characteristic views of the complex. With that knowledge, ab initio reconstruction was performed in cryoSPARC followed by 7 iterations of homogeneous refinement using D2 symmetry. We found that particles picked using our method yield a structure at 3.0 Å resolution determined using the 0.143-cutoff of the FSC curve, compared to 3.1 Å obtained from Topaz. While our method results in almost 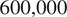 more particles compared to Topaz, the resolution difference is not significant, indicating that factors other than the number of particles may be limiting the resolution in this case. In order to further assess the quality of the particles, we removed particles identified using Topaz from the set of particles obtained using our method, and used these particles to produce a 3D reconstruction. Using the resulting 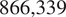 particles (picked by our method and missed by Topaz), we obtained a 3D structure at 3.15 Å (Figure 6). This validates the quality of the set of additional particles identified using our method, showing that they can be used to produce a reconstruction of similar resolution to that obtained using Topaz particles only.

#### 4.3.4. EMPIAR-10304

We manually labeled 12 tilt series and treated manual labels as the ground truth. Precision, recall, and F1 scores are calculated as described in Equation 15. Using a radius of three pixels, our method obtains a precision of 0.70 and recall of 0.86, compared to 0.48 and 0.67 obtained from Topaz picking, and 0.34 and 0.22 obtained from crYOLO. Similar to the K63 ribosome dataset, we observed a lack of generalizability of Topaz and crYOLO to unseen data distributions.

## 5. Discussion

In this section, we delve into a deeper analysis of the proposed joint-learning framework. Specifically, we look at how denoising can be beneficial to detection, how to choose the proper positive prior 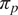 , and analyze the effect of hyperparameters on overall performance.

### 5.1. Features learned through denoising can be directly used for detection

**Figure 7.**
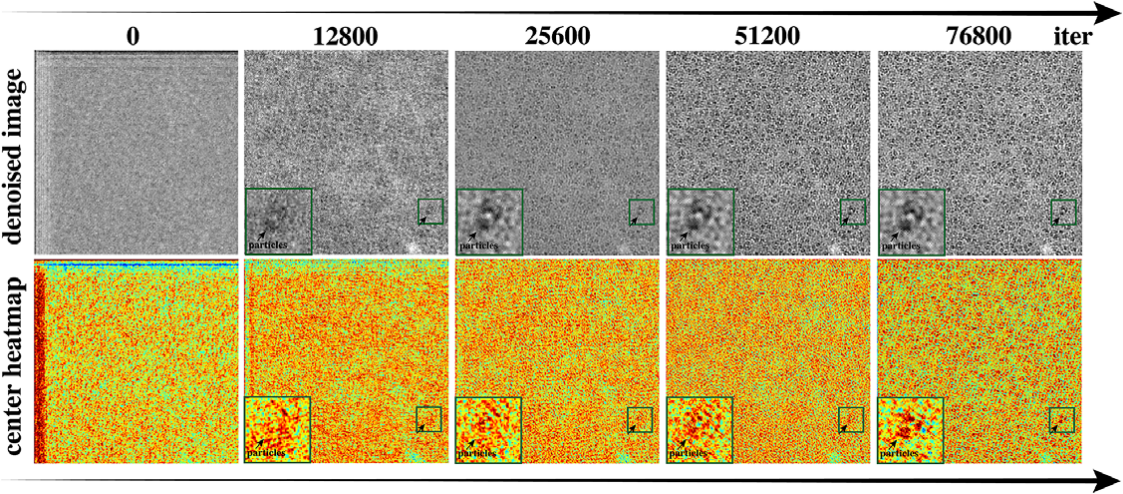
**Progression of denoised images and detection heatmaps during training.** We show outputs from the 0th (untrained, random initialization); 12,800th; 25,600th; 51,200th; and 76,800th iterations. When training using the denoising loss alone, as the quality of the denoised output improves during training, it is easier to differentiate particle locations from the background on the detection heatmap (black rectangles), even though the detection head remains untrained. This shows that features learned through denoising are sufficient for the detection task without explicit detection training.

In order to better understand how denoising can be beneficial to detection, we froze the weights of the detection head, trained the model with denoise loss exclusively (without the PU loss for detection), and assessed the resulting particle center heatmap generated by the untrained detection head (the input features to the detection head are trained through denosing loss only). The model received inputs consisting of both particle-containing patches and random patches cropped from the input micrograph. As Figure 7 suggests, at the beginning of training, where all weights are randomly initialized, it is very hard to separate particles from the background using the resulting heatmap. As weights are updated based on the denoising loss, input features to the detection head are no longer random. Therefore, even though the weights of the detection head remain random, by using learned features, it is easier to differentiate particles from the background on the generated output. This shows that with denoising, the background and particle features become more easily separable. This separability improves particle detection accuracy. This also means that features extracted through denoising learning are good enough for particle detection and additional feature extraction is not necessary. Therefore, instead of performing denoising and detection independently where features are learned multiple times, these two tasks can be performed jointly as the same set of features can be used to perform both tasks. In addition, as shown in Table 1, joint learning leads to better denoising results compared to performing denoising alone.

### 5.2. Prior belief and its effect on performance

As particles are identified on a per-pixel basis, even for crowded datasets, we use a relatively small class prior probability 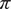 . For CnTPS, EMPIAR-10215, and EMPIAR-10304, we used 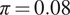 , and for the K63 ribosome, we used 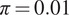 . This is because the actual number of pixels that belong to the center of particles is relatively small compared to the total number of pixels in a micrograph. If we use a larger positive prior, more FPs get identified and the resulting heatmaps tend to have higher scores. Similarly, if we use smaller positive priors, we are able to maintain a high true-positive rate while sacrificing the rate of FNs. While having a reliable estimation of 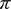 is important, this can be obtained by simple visual inspection of micrographs and the size of the particles when doing annotations. For instance, 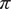 can be estimated by calculating the product of the expected number of particles per micrograph by the number of pixels per particle, and then dividing this product by the total number of pixels in a micrograph.

### 5.3. Effects of other hyperparameters on performance

In order to assess how hyperparameters in the loss function (Equation 14) can affect the performance of our algorithm, we performed experiments with 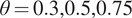 , and 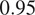 . We found that as 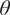 decreases, the performance of both denoising and detection tasks worsens. This makes sense as while information provided by both tasks is complementary, the detection task is more dependent upon the denoising task: information from the detection task can be used to refine learned features from the denoising task, instead of generating those features (as suggested in the previous section). Therefore, the denoising task should be the dominating task in this case. The choice of 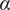 and 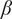 depends on the SNR level of the datasets. For datasets with lower SNR where proteins are harder to identify, 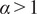 results in better performance. Increasing 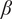 forces pixels located at the center of particles to have a higher detection probability (classified as particle) and pixels around the center of particles to have a lower probability (classified as background). Therefore, for well-annotated training data, increasing 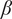 leads to better center localization. Experimentally, we found that 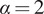 and 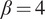 works for most cases. The consistency regularization weight 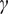 has only minor effects on the overall performance.

## 6. Conclusion

Even though many deep-learning-based denoising and particle picking methods have been proposed, existing methods either require a large amount of training data or additional manual paired image curation, and most importantly, they yield unsatisfying results on extremely low SNR and crowded datasets. In these cases, the use of denoising as a preprocessing step can aid the detection task. However, this step takes additional time, and in many cases, naive denoising can lead to over-smoothing, which leads to a high FN rate. Intuitively, micrograph denoising and particle identification should not be treated as two completely different problems. Instead, these two tasks are complementary: the underlying data distribution for background and particles are different, and by knowing the location of particles, we should be able to produce a denoised micrograph that better differentiates particles from background. Similarly, with cleaner micrographs, we should be able to identify particles more easily. Therefore, by enabling information sharing through joint training, we expect the performance of both tasks to improve. Here, we present a framework that performs micrograph denoising and particle detection simultaneously without the need of noiseless images or fully annotated datasets. The framework is able to identify small particles under low SNR conditions and enables better visualization of these particles in both single-particle cryo-EM and CET images. In addition, this approach can be easily adapted to perform a single task if only one of the tasks is needed.

Even though the proposed framework uses a dataset-specific model, training requires only a few partially annotated micrographs with a total of 100+ particle annotations, which significantly reduces manual labor and training time. In addition, as shown in Reference (3), dataset-specific models often outperform generalized particle picking models, especially in the case of challenging datasets. Similar to Reference (3), our method requires only one hyperparameter, the class prior, which can be estimated by visual inspection of micrographs during data labeling. While the default value for the task weight ( 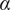 ) works well on multiple datasets, further dataset-specific tuning can further improve the performance.

Our joint framework offers a viable way to enable particle localization under some of the most challenging scenarios, such as those encountered when imaging proteins with low-molecular weight or in crowded environments. The proposed method is modular, which allows it to be easily incorporated into existing data processing software and is currently integrated into nextPYP.^(^^62^^)^ A pre-trained model for micrograph denoising can be downloaded from Zenodo. In addition, the framework can be extended to the preprocessing of CET tilt series to improve tilt series alignment and tomogram reconstruction. We hope that our algorithm will facilitate and expedite the structural analysis of challenging biomolecular targets such as low-molecular-weight complexes or thick specimens imaged in their native context.

## Data Availability

The source code is freely available from https://github.com/nextpyp/spr_pick. A pre-trained model for the denoising module is available at https://doi.org/10.5281/zenodo.10445797.
